# Long-term menopause exacerbates vaginal wall support injury in ovariectomized rats by regulating amino acid synthesis and glycerophospholipid metabolism

**DOI:** 10.3389/fendo.2023.1119599

**Published:** 2023-06-22

**Authors:** Xia Yu, Li He, Wenyi Lin, Xuemei Zheng, Ling Zhang, Bo Yu, Yanjun Wang, Zhenglin Yang, Yonghong Lin

**Affiliations:** ^1^ Department of Clinical Laboratory, Chengdu Women’s and Children’s Central Hospital, Sichuan Provincial People’s Hospital, School of Medicine, University of Electronic Science and Technology of China, Chengdu, Sichuan, China; ^2^ Department of Obstetrics and Gynecology, Chengdu Women’s and Children’s Central Hospital, School of Medicine, University of Electronic Science and Technology of China, Chengdu, Sichuan, China; ^3^ Department of Medical Pathology, Chengdu Women’s and Children’s Central Hospital, School of Medicine, University of Electronic Science and Technology of China, Chengdu, Sichuan, China; ^4^ Sichuan Provincial Key Laboratory for Human Disease Gene Study and Institute of Laboratory Medicine, Sichuan Provincial People’s Hospital, University of Electronic Science and Technology of China, Chengdu, China

**Keywords:** pelvic organ prolapse, menopause, vaginal wall, mechanical properties, transcriptomic, metabolomic

## Abstract

**Purpose:**

Menopause is a risk factor for pelvic organ prolapse (POP) and is frequently associated with diminished vaginal wall support. To uncover relevant molecular mechanisms and provide potential therapeutic targets, we evaluated changes in the transcriptome and metabolome of the vaginal wall in ovariectomized rats to identify important molecular changes.

**Methods:**

Sixteen adult female Sprague−Dawley rats were randomly assigned to either the control or menopause group. Seven months after the operation, hematoxylin and eosin (H&E) staining and Masson trichrome staining were used to observe changes in the rat vaginal wall structure. Differentially expressed genes (DEGs) and metabolites (DEMs) in the vaginal wall were detected by RNA-sequencing and LC−MS, respectively. Gene Ontology (GO) and Kyoto Encyclopedia of Genes and Genomes (KEGG) analyses of DEGs and DEMs were performed.

**Results:**

We verified that long-term menopause causes vaginal wall injury by H&E and Masson trichrome staining. From the multiomics analyses, 20,669 genes and 2193 metabolites were identified. Compared with the control group, 3255 DEGs were found in the vaginal wall of long-term menopausal rats. Bioinformatics analysis showed that the DEGs were mainly enriched in mechanistic pathways, including cell−cell junction, extracellular matrix, muscle tissue developments, the PI3K-Akt signaling pathway, the MAPK signaling pathway, tight junctions and the Wnt signaling pathway. Additionally, 313 DEMs were found, and they consisted mostly of amino acids and their metabolites. DEMs were also enriched in mechanistic pathways, such as glycine, serine and threonine metabolism, glycerophospholipid metabolism, gap junctions and ferroptosis. Coexpression analysis of DEGs and DEMs revealed that biosynthesis of amino acids (isocitric acid and *PKM*) and glycerophospholipid metabolism (1-(9Z-hexadecenoyl)-sn-glycero-3-phosphocholine and *PGS1*) are critical metabolic pathways, suggesting that POP induced by menopause may be associated with the regulation of these processes.

**Conclusion:**

The findings showed that long-term menopause greatly exacerbated vaginal wall support injury by decreasing the biosynthesis of amino acids and interfering with glycerophospholipid metabolism, which may result in POP. This study not only clarified that long-term menopause exacerbates damage to the vaginal wall but also provided insight into the potential molecular mechanisms by which long-term menopause induces POP.

## Introduction

Pelvic organ prolapse (POP) is a common pelvic floor dysfunction (PFD) that lowers the quality of life for almost half of all women globally. Increased life expectancy and efforts to improve quality of life have led to an increase in both the prevalence of POP and the number of women seeking medical attention for their symptoms, resulting in increased financial and medical costs (1). Between 2010 and 2050, the number of women in the United States who have POP is anticipated to rise by 46%, from 3.3 million to 4.9 million (2). In the United States, 10% - 20% of women have pelvic floor reconstruction surgery to cure prolapse (3); however, complications such mesh exposure and recurrence can still occur (4). Therefore, exploring the mechanism of action of POP is crucial for the development of new therapeutic strategies.

Postmenopausal, low-estrogen environments are a risk factor for the development of POP (5). As age increased from 20-29 years old to over 50 years old, the likelihood of experiencing symptomatic POP also increased. The odds ratio for symptomatic POP-Q stage II or higher rose from 1.34 (95% CI, 1.32-1.45) to 7.34 (95% CI, 4.34-12.41) (6). And the prevalence of symptomatic POP is 30% - 40% in postmenopausal women (5). According to these findings, the researchers further conducted estrogen supplementation therapy for postmenopausal patients with POP, but the clinical therapeutic effect remains controversial (7, 8). Therefore, it is urgent to elucidate the molecular mechanism of POP induced by low estrogen environment in order to find therapeutic targets.

Multiomics analysis methods can be applied to clarify the pathophysiology of disease and the underlying mechanisms (9). Moreover, gene expression profiles for the entire genome can be provided through transcriptomics. Metabolomics offers large-scale informative data about metabolic changes that reflect genetic, epigenetic, and environmental influences on cellular physiology. Transcriptomics and metabolomics have recently been employed to investigate the postmenopausal, low estrogen environment. Lu et al. found that the metabolic pathways of patients with premature ovarian insufficiency were abnormal, and some metabolites, such as fumarate, arachidonic acid and acetoacetic acid, were altered in the patient’s serum. The DEGs that were found in patients with premature ovarian insufficiency were mainly associated with extracellular matrix (ECM)/structural organization, different types of junctions, and various catabolic/metabolic/biosynthetic processes. In addition, cytokine−cytokine receptor interactions, the PI3K-Akt signaling pathway, and the MAPK signaling pathway was also enriched, which have close regulatory effects on cellular mechanical properties (10, 11). Zhu et al. demonstrated that abnormal ECM synthesis and metabolism affected the pelvic support system, which led to the occurrence and progression of POP (12). Moreover, in our recent studies, we constructed focal adhesion signaling pathway-related ceRNA networks in POP by transcriptome analysis, which were found to be associated with mechanical properties (13). We also revealed that abnormal metabolic regulatory pathways and gene transcription expression were closely related to the occurrence of POP on the uterosacral ligaments (14). However, the genetic transcription and metabolic effects of prolonged menopause on the vaginal wall tissue, an important part of the pelvic floor support structure, have not been elucidated.

In this study, to address these issues, we constructed an animal model of POP by removing both ovaries in rats and used a postoperative care period of up to seven months to create a long-term menopausal environment, followed by a multiomic analysis of the rat vaginal wall tissue including transcriptomics and metabolomics. To our knowledge, this is the first study to use a multiomics analysis to investigate the specific mechanism by which prolonged menopause exacerbates support damage to the vaginal wall in rats and to provide targets that may interfere with the occurrence of POP.

## Materials and methods

### Animals and ovariectomy surgery

Female Sprague−Dawley rats weighing between 190 and 220 g and two months old were purchased from the Chengdu Dashuo Laboratory Animal Co., Ltd. Individually housed rats were kept in a specific pathogen-free environment with free access to food and water in a 12:12 light:dark cycle and a temperature range of 22–26°C and humidity range of 45–55%. The ethics committee of the Chengdu Women’s and Children’s Central Hospital approved the use of lab animals for our research objectives in accordance with the guidelines from the Animal Experimental Center.

**Figure 1:**
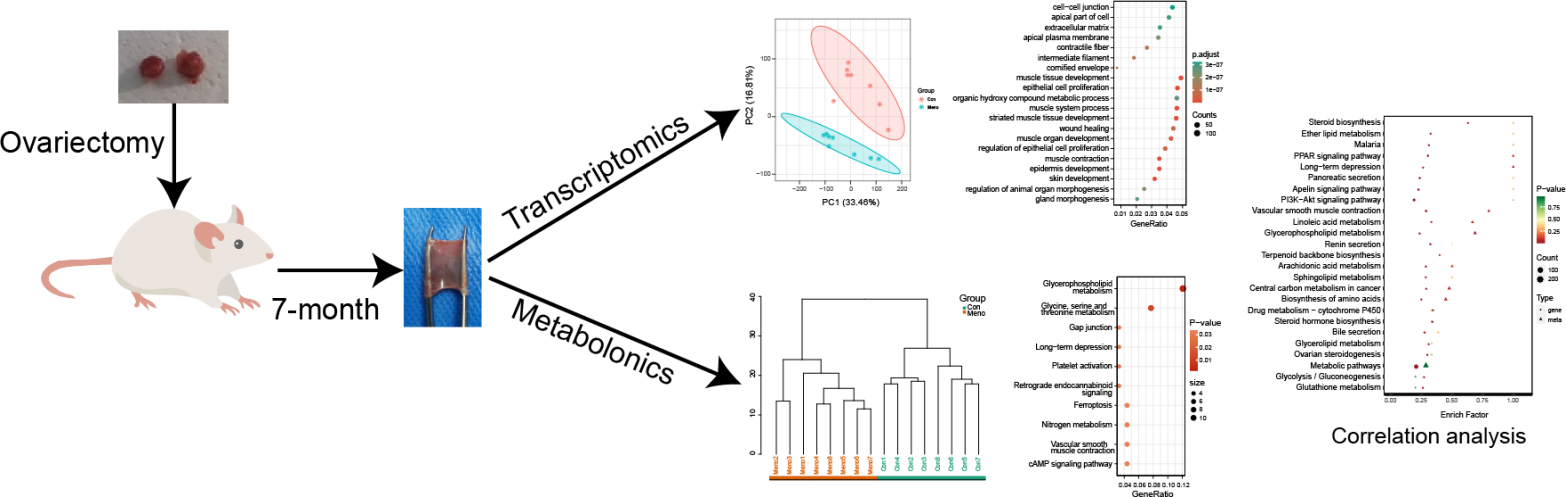
The research strategy adopted in the present study.

The rats were randomly assigned to either the control group (Con, n=8) or the menopausal group (Meno, n=8) after a week of acclimation. An intraperitoneal injection of 1% pentobarbital sodium (0.4 mL/100 g) was used to anesthetize the rats. A 2-cm ventral midline incision was performed in the upper abdomen while the area was sterile. Bilateral ovaries were removed from the Meno group. The ovaries in the control group were exposed but not removed. After seven months of postoperative care, as shown in **Figure 1**, the rats were sacrificed.

### Sample collection

The rats were sacrificed, and whole blood retrieved by direct cardiac puncture was used to measure serum estrogen. Approximately 4 mm of vaginal wall tissue close to the cervix was incised, fixed in 10% neutral buffered formalin and embedded in paraffin for histological tests. Transcriptomics and metabolomics investigations were conducted on the remaining tissue. Prior to analysis, each sample was kept at -80°C.

### Serum estrogen measurements

Following the ovariectomy, the serum E2 levels were measured seven months later through enzyme-amplified chemiluminescence using the collected blood samples (Atellica IM1600, Siemens). Estradiol and progesterone assay analytical sensitivity limits were 15 pg/mL (references range of 20-2000 pg/mL).

### H&E and Masson trichrome staining

For the morphological analysis, a 3-µm thick cross-section of a paraffin-embedded vaginal wall tissue from each group was stained with Masson trichrome and H&E. The samples were scanned using the K-Viewer (1.7.0.23) X64 digital pathology scanning system at magnifications of ×4 and ×10.

### Transcriptome sequencing and data analysis

Total RNA was extracted from the samples using TRIzol reagent (Invitrogen, USA). An Agilent 5400 system (Agilent Technologies, USA) was used to measure RNA concentrations and integrity. The RNA sample preparations used a total of 1 g of RNA per sample as input material. Metware Biotechnology Co., Ltd. (Wuhan, China) prepared the cDNA libraries for sequencing on the Illumina Novaseq 6000 using the NEBNext^®^Ultra RNA Library Prep Kit. To filter the original data, fastp v 0.23.2 was used. Reads with adapters, sequences with more than 10% unknown nucleotides (N), and a quality rating of less than 50% (Q-value ≤20) were removed.

Clean reads served as the foundation for all further analyses. HISATv2.1.0 software was used to build the index, download the reference genome Rnor6.0.103 (http://ftp.ensembl.org/pub/release-103/fasta/rattusnorvegicus/) and associated annotation files, and compare clean reads to the reference genome. The length of each gene and the number of reads mapped to it were used to compute the FPKM of each gene. Using DESeq2 software (v1.22.1), the differential expression between the two groups was examined. DEGs were defined as genes with |log2FC| > 1 and q-value < 0.05. We used KEGG pathway analysis and GO functions to estimate the potential biological roles of the DEGs, the clusterProfiler R package (v3.14.3) was employed.

### Metabolite profiling analysis

MetWare (Wuhan, China) carried out the extract analysis, metabolite identification, and quantification in accordance with their established practices and earlier research [14]. After the raw data had been processed, the unique metabolites for the two-group analysis were identified using the absolute VIP (VIP ≥ 1), *P*-value (*p* < 0.05, Student’s t test), and absolute Log2FC (|Log2FC| ≥ 1.0) values.

### Coexpression network analysis of the metabolome and transcriptome

According to the fold changes of each DEG and each DEM, the Pearson correlation coefficients were calculated using the EXCEL application. Correlations with coefficients of R^2^ > 0.8 were chosen.

## Results

### Estrogen concentrations decrease during prolonged menopause

**Figure 2:**
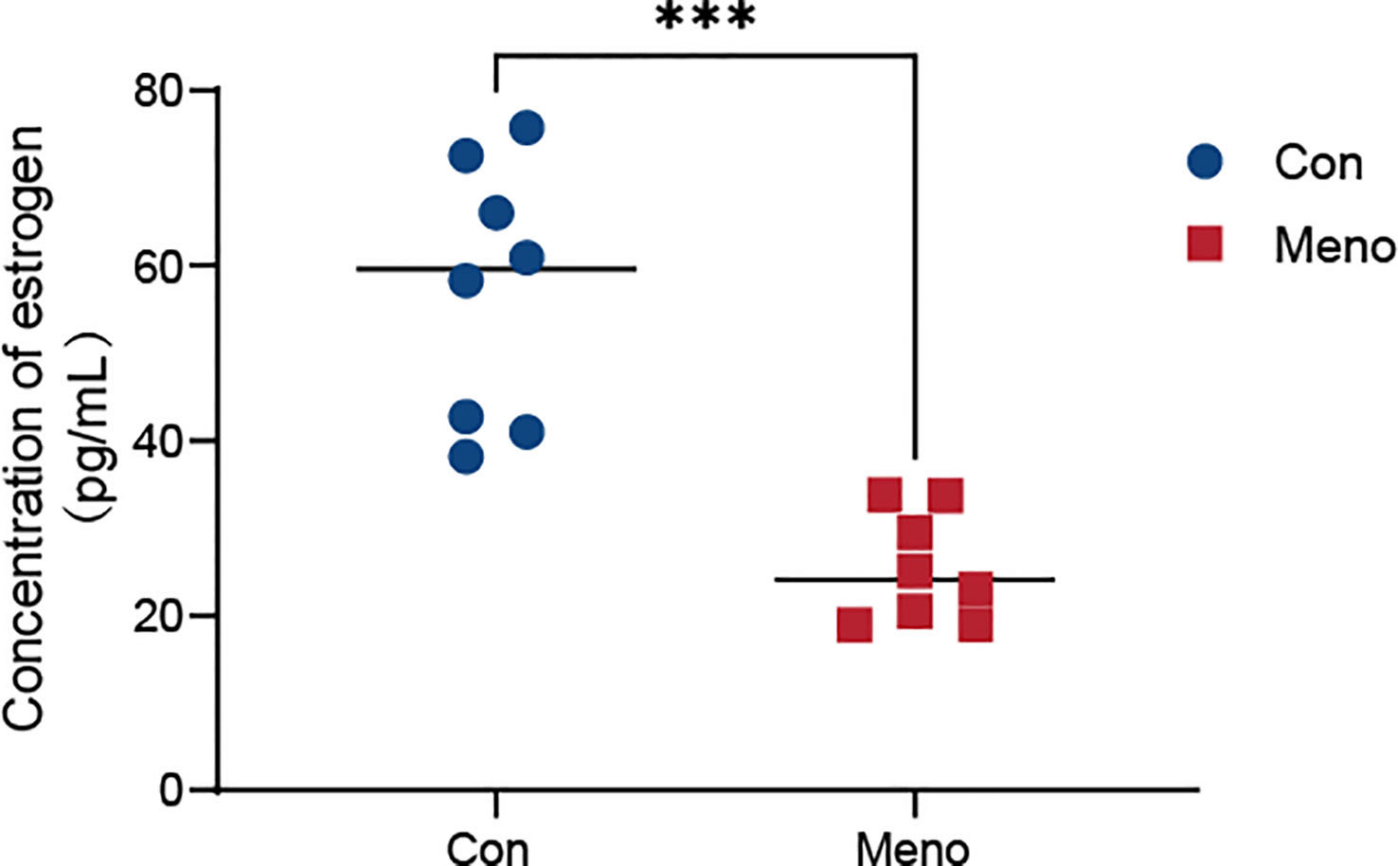
Serum estrogen levels in the Con group and the Meno group (n=8, ***P < 0.001).

To verify the effect of ovariectomies on estrogen levels, we analyzed serum estrogen concentrations in rats seven months after ovariectomies by chemiluminescence. The blood levels of estrogen in the control group fluctuated within a normal range (38.13 pg/ml - 75.74 pg/ml), but seven months after ovariectomy, they plummeted to a low level (18.86 pg/ml - 33.74 pg/ml), and there was a significant difference between the two groups (*p* < 0.001) (**Figure 2**). Therefore, vaginal wall tissues from the menopause group and the control group seven months after surgery were selected as material samples for transcriptomic and metabolomic analysis.

### Histological staining revealed abnormal vaginal wall tissue structure

**Figure 3:**
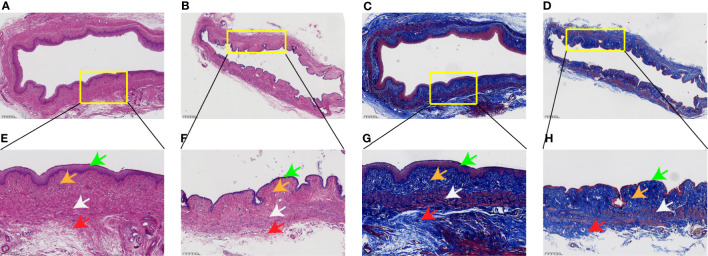
Rat vaginal wall tissue stained with H&E and Masson’s trichrome. H&E staining of the Con group **(A)** ×4, **(E)** ×10, and Meno group **(B)** ×4, **(F)** ×10. Masson’s trichrome staining of the Con group **(C)** ×4, **(G)** ×10, and Meno group **(D)** ×4, **(H)** ×10. Epithelial layer (green arrows), lamina propria (orange arrows), muscle layers (white arrows) and adventitia layer (red arrows).

As shown in **Figure 3**, H&E and Masson’s trichrome staining were used to evaluate the histology of the vaginal wall and to examine the histopathological changes in the tissue after long-term menopause. In the control group, the vaginal epithelial layer was abundant, collagen deposition was dense in the lamina propria, the normal orderly arrangement of smooth muscle layers was maintained, the muscle cells were clearly outlined, and there was a thick adventitia layer. However, in the Meno group, histological analysis showed a significant reduction in the thickness of the vaginal epithelial layer, a sparse state of collagen deposition in the lamina propria, a disordered arrangement of smooth muscle with a significantly reduced content, and the adventitia layer became thinner. Therefore, long-term menopause weakens the tissue structure of the vaginal wall, resulting in a decline from the epithelial layer to the adventitia layer, which exacerbates the decline in the support capacity of the vaginal wall.

### Transcriptome analysis reveals altered molecular expression of vaginal wall tissue in rats after long-term menopause

**Figure 4:**
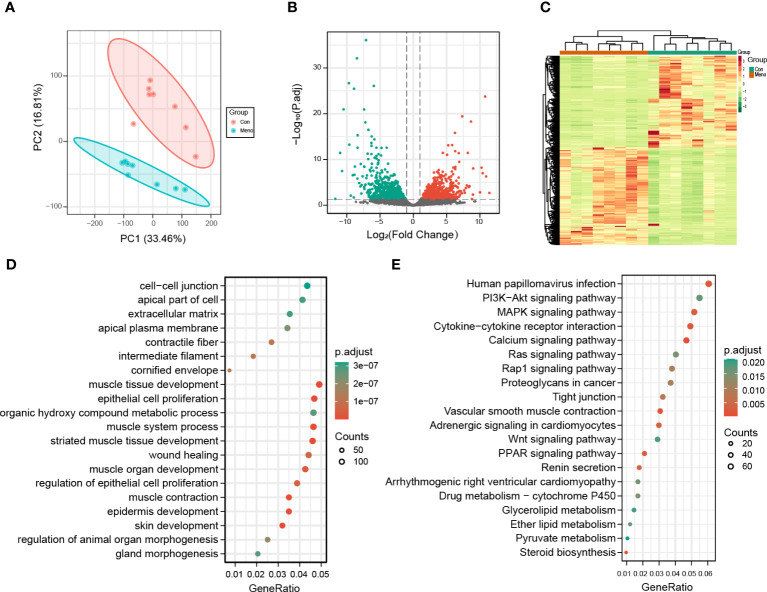
Transcriptomic analysis of the Con and Meno groups. **(A)** PCA plots of the two groups. **(B)** Volcanic distribution maps of the Con group and Meno groups. **(C)** Heatmaps of significant differentially expressed genes. **(D-E)** Bubble plots showing GO and KEGG enrichment analysis pathways of differentially expressed genes.

To explore the molecular events occurring in the vaginal wall of long-term postmenopausal rats, transcriptome analysis was performed on vaginal wall tissue in rats seven months after ovary removal (Meno) and without ovary removal (Con). After removing the low-quality reads, a total of 721,846,394 clean reads were obtained. The percentages of Q30 and GC were 90.94–92.02% and 47.86–50.59%, respectively, indicating that the transcriptome sequencing data was high quality. The PCA results showed that the transcriptome of the Meno group was very different from that of the control group (**Figure 4A**). A total of 20,669 genes were functionally annotated in the databases (**Additional file 1**). Moreover, 3255 (1677 up- and 1578 down-regulated) DEGs (|fold change| > 2 and q-value < 0.05) were identified in the comparison of Con vs. Meno (**Figures 4B, C**) (**Additional file 2**). Overall, the results suggested that long-term menopause can induce transcriptional changes in the vaginal parietal tissue. The raw data can be found at https://www.ncbi.nlm.nih.gov/geo/query/acc.cgi?acc=GSE220515.

### Functional analysis of DEGs in pathways related to mechanical properties

To further evaluate the biological functions of the DEGs in the vaginal wall tissue of long-term menopausal rats, we performed Gene Ontology (GO) and Kyoto Encyclopedia of Genes and Genomes (KEGG) enrichment analyses (**Additional File 3**). The results showed that the GO terms significantly enriched by the DEGs in the Meno group vs. the Con group were closely related to mechanical properties, including cell−cell junction, apical part of cell, extracellular matrix, muscle tissue developments, and epithelial cell proliferation (**Figure 4D**). The KEGG pathway analysis of the DEGs in the Meno group vs. the Con group also exhibited signaling pathways related to mechanical properties, including the PI3K-Akt signaling pathway, the MAPK signaling pathway, cytokine−cytokine receptor interaction, calcium signaling pathways, tight junctions and the Wnt signaling pathway (**Figures 4E**). Therefore, the results showed that long-term menopause can regulate complex biological pathways related to the mechanical support of vaginal wall tissue.

### Metabolomic analysis reveals disturbances in amino acid synthesis and metabolites associated with mechanical pathways

**Figure 5:**
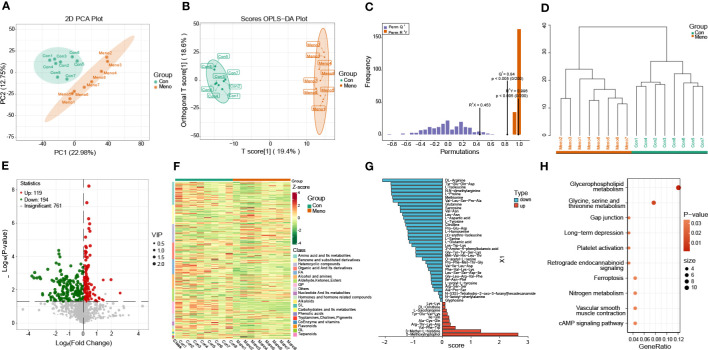
Metabolomics analysis of the Con and Meno groups. **(A)** PCA plot of the two groups. **(B)** The OPLS-DA score plot and **(C)** OPLS-DA model test chart demonstrated favorable discrimination between the Con and Meno groups, R^2^X=0.453, R^2^Y=0.996, Q^2 =^ 0.84, *p* < 0.05. **(D)** Cluster analysis plot of differentially expressed metabolites (DEMs) in the Con and Meno groups. **(E, F)** Volcano map and heatmap of the DEMs in the Con vs. Meno groups. **(G)** OPLS-DA model shows fold changes in the expression of amino acids and their metabolism between the two groups. **(H)** Bubble plot showing pathway enrichment analysis related to DEGs in the Con and Meno groups.

For further analysis of metabolites of long-term menopausal rat vaginal wall tissue, untargeted (global) metabolomics using liquid chromatography high-resolution mass spectrometry (LC−MS) was carried out to obtain a comprehensive comparative analysis of metabolites. A total of 2193 metabolites were obtained in all samples (**Additional File 4**) and clearly divided into two groups based on PCA (**Figure 5A**). The orthogonal partial least squares discriminant analysis (OPLS-DA) model was then used to further investigate the DEMs between the groups. The OPLS-DA results show that the Meno and Con groups are scattered in two different geographic regions (**Figure 5B**). The OPLS-DA model has a satisfactory fit, strong predictive power, goodness-of-fit and predictive power values (R^2^X = 0.453, R^2^Y = 0.996, Q^2^ = 0.84, *p* < 0.005) (**Figure 5C**). We also carried out cluster analysis on 16 samples. The samples that clustered together showed higher similarity among the samples, as shown in **Figure 5D**. Based on the VIP > 1 and *P value* < 0.05, the number of upregulated and downregulated metabolites in the Meno group vs. the Con group was 119 and 194, respectively (**Figure 5E**) (**Additional File 5**). The was nearly perfect separation between the Meno group and the Con group. Then, these DEMs were summarized into 20 groups, mainly divided into amino acids and their metabolites (46), benzene and substituted derivatives (38), heterocyclic compounds (37), organic acids and their derivatives (33), FA (30), alcohols and amines (28) and others (**Figure 5F**). After qualitative and quantitative analysis of the detected amino acids and their metabolites, we combined the groups and showed the differential fold changes between amino acids and their metabolites in the two groups, as shown in **Figure 5G**. The histogram showed that the expression of more than 30 amino acids and their metabolites in the Meno group were reduced compared to those in the Con group. This suggests that prolonged menopause leads to a significant decrease in amino acid synthesis. Similarly, the top 10 KEGG enrichment analyses showed that the DEMs were associated with glycine, serine and threonine metabolism (**Figure 5H**) (**Additional File 6**). In addition, the most obvious pathway of DEM enrichment was glycerophospholipid metabolism, which is closely related to cellular mechanical properties. Moreover, the DEMs were also enriched in other pathways associated with mechanical properties, including gap junctions and ferroptosis. The results showed that long-term menopause can induce changes in metabolites involved in cellular mechanical properties.

### Coexpression analysis of DEGs and DEMs revealed that the decreased mechanical support of the vaginal wall was closely related to biosynthesis of amino acids and glycerophospholipid metabolism

**Figure 6:**
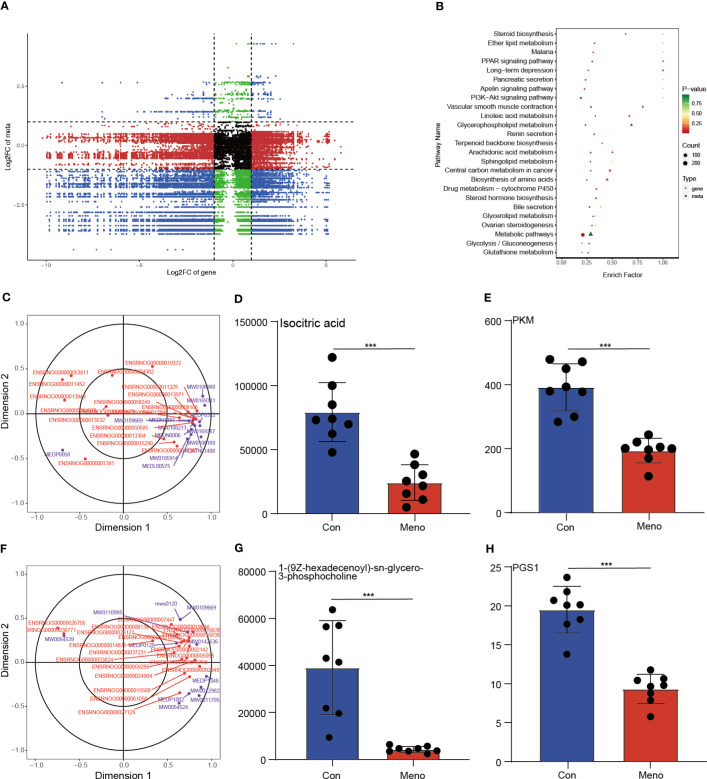
Integration of the transcriptome and metabolomic data analysis. **(A)** The nine-quadrant plot shows the coexpression analysis of DEGs and DEMs. **(B)** The bubble chart shows the common enriched pathways of DEGs and DEMs. Canonical correlation analysis (CCA) results reveal the role of DEGs and DEMs in amino acid biosynthesis **(C)** and glycerophospholipid metabolism **(F)**. The histogram shows the expression levels of isocitric acid **(D)**, *PKM* **(E)**, 1-(9Z-hexadecenoyl)-sn-glycero-3-phosphocholine **(G)** and *PGS1* **(H)** in the Con and Meno groups (n=8, ***P < 0.001).

To investigate the relationship between the DEGs and DEMs in the vaginal wall tissues of rats experiencing long-term menopause, a coexpression analysis was carried out and displayed by a nine-quadrant plot (**Figure 6A**) (Pearson correlation coefficient > 0.8, *p* < 0.05; **Additional File 7**). The lower left quadrant results showed that the proportion of positively correlated substances with decreased gene expression and decreased metabolite expression accounted for most of the DEGs and DEMs. The KEGG pathway coenriched by the two omics was used to draw the bubble map, and the results showed that the DEGs and DEMs were significantly enriched in biosynthesis of amino acids and glycerophospholipid metabolism (**Figure 6B**). Then, the results of a canonical correlation analysis (CCA), a multivariate statistical analysis technique that uses the correlation between pairs of comprehensive variables to reflect the overall correlation between two groups, revealed DEGs and DEMs play a role in the biosynthesis of amino acids and glycerophospholipid metabolism (**Figures 6C, F**). In addition, the role of DEGs and DEMs in the biosynthesis of amino acids and glycerophospholipid metabolism was further investigated and significantly correlations were found between isocitric acid and *PKM*, 1-(9Z-hexadecenoyl)-sn-glycero-3-phosphocholine and *PGS1*, respectively. Therefore, we further investigated the expression of isocitric acid, *PKM*, 1-(9Z-hexadecenoyl)-sn-glycero-3-phosphocholine and *PGS*, and their expression was reduced in the long-term menopausal state (**Figures 6D, E, G, H**). The integrated analysis of transcriptomics and metabolomics further revealed that the decreased mechanical support of vaginal wall tissue in rats during long-term menopause was closely related to the biosynthesis of amino acids and glycerophospholipid metabolism.

## Discussion

In this study, to explore the impact of long-term menopause on POP and to provide possible therapeutic targets, we used a comprehensive analysis of transcriptomics and metabolomics to investigate the changes in the vaginal wall in a rat model with bilateral ovary removal after a postoperative care period of seven months. The results showed that long-term menopause can exacerbate the decreased vaginal wall support, mainly through interference with the biosynthesis of amino acids and glycerophospholipid metabolism, which are involved in the occurrence of POP.

To determine the successful establishment of a POP animal model by bilateral ovariectomy in rats, serum estrogen levels were measured and the pathological structure of the rat vaginal wall tissue was detected. Consistent with previous reports, the serum estrogen level in rats seven months after bilateral oophorectomy was significantly lower than that of the control group (15), and the structure of the vaginal wall was also abnormal. The pathological results showed that long-term menopause resulted in thinning of the vaginal wall (16). Moreover, a sparse state of collagen deposition and a disordered arrangement of smooth muscle with a significantly reduced content were found. According to a report on the biaxial mechanical response, these changes in the vaginal wall reduced maximum muscle tone (17).

To investigate the potential mechanism by which prolonged menopause affects the mechanical properties of the vaginal wall, we compared the transcription profiles of the Con group and Meno group vaginal wall tissues to identify DEGs. GO and KEGG pathway enrichment analyses indicated that these DEGs had notable correlations with cellular mechanical pathways, including extracellular matrix, cell−cell junctions, muscle tissue developments, the PI3K-Akt signaling pathway, the MAPK signaling pathway, tight junctions and the Wnt signaling pathway. The ECM is a highly dynamic structure that is present in all tissues and continuously undergoes controlled remodeling. In addition to serving as a structural support for tissue elasticity and integrity, the ECM also functions to communicate mechanical signals to cells that control adhesion, migration, proliferation, and apoptosis within the matrix. These signals are transmitted by ECM constituents acting as ligands for cell receptors such as integrins (18). Several studies have reported the use of ECM remodeling to construct animal models of POP (19–21) and the selection of ECM remodeling as a therapeutic target (22). Moreover, the site of cell-ECM adhesion is limited by the application of fibronectin micropatterns. According to Théry et al., the ECM has an impact on the strength of intra- and intercellular forces as well as the stability of cell−cell junction placement (23). The tight junction and cell−cell junction molecular landscapes are diverse, containing transmembrane proteins that form intercellular bonds and a variety of cytoplasmic proteins that remodel the junctional connection to the cytoskeleton and transform mechanical cues into cellular responses (24), which involve the PI3K-Akt signaling pathway (25), MAPK signaling pathway (26) and Wnt signaling pathway (27). In addition, using single-cell RNA-seq analysis, Zhu et al. also showed dysregulation of the ECM and cell-cell communication patterns in the prolapse of the anterior vaginal wall (28). In POP samples, the interactions of smooth muscle cells with fibroblasts and macrophages were increased, which may affect tissue regeneration, such as muscle tissue development and matrix organization. In addition, in the process mechanical transduction pathway signaling, the metabolism of substances is an important biological process for the production of biosynthetic macromolecules (29). Additionally, Alarab et al. discovered that menopause affects the genes involved in the metabolism of the vaginal extracellular matrix (30). The unnatural accumulation of numerous significant metabolites that profoundly affected cellular function was caused by disorganized metabolic processes.

Herein, untargeted metabolomics was used to further investigate the decreased mechanical support of the vaginal wall in rats with prolonged menopause. From our data, the expression of more than 30 amino acids and their metabolites in the Meno group was reduced compared to that in the Con group, and DEMs were also mainly enriched in cellular mechanic-related pathways, such as glycine, serine and threonine metabolism, glycerophospholipid metabolism, gap junctions and ferroptosis. Collagen, the most abundant protein in the body, is necessary to preserve the ECM’s structure and strength, and glycine, proline, and hydroxyproline (Hyp) make up 57% of t collagen’s overall amino acid content (AAs) (31). Long-term menopause decreases the expression of amino acids and their metabolites, especially those involved in glycine, serine and threonine metabolism, which are closely related to ECM remodeling and leads to gap junction disturbances. Glycerophospholipid metabolism is closely related to the formation of cell membranes, in which many biological processes take place through mechanical signaling (32). Ferroptosis is closely associated with amino acid, lipid, and iron metabolism. The formation of cell−cell contacts in epithelial cell monolayers is thought to improve resistance to ferroptosis induction (33). Our findings show significant thinning of the vaginal epithelial layer after long-term menopause; however, more studies are needed to confirm whether this change promotes ferroptosis and leads to POP. At present, this is the first study to suggest that ferroptosis may be related to the occurrence of POP.

Furthermore, this study integrated transcriptome and metabolome data from our biological model, and revealed a significant correlation between the DEGs and DEMs that is associated with amino acid biosynthesis and glycerophospholipid metabolism. Additionally, isocitric acid and *PKM*, 1-(9Z-hexadecenoyl)-sn-glycero-3-phosphocholine and *PGS1* were found to have the highest correlation in these pathways. Yan et al. reported that modulation of *PKM* splicing inhibited hepatocyte metabolic reprogramming and could be a target for the treatment of hepatocellular carcinoma (34). However, the *PKM* as a target for the treatment of POP needs to be further explored. In our previous studies of the uterosacral ligaments in patients with POP, we also reported that *PGS1* was involved in glycerophospholipid metabolism alterations (14). The uterosacral ligaments and the vaginal wall are both important tissues that make up the pelvic floor support, and abnormal expression of *PGS1* was found in both of these tissues, so *PGS1* may be an important target for the treatment of POP.

Although this study found the DEGs, DEMs and related enrichment pathways that were involved in the decreased vaginal wall support in rats during long-term menopause, this study has limitations. First, the animal model of POP created by bilateral ovariectomy of rats does not accurately represent the characteristics of clinical POP patients. Follow-up studies require the collection of tissue from a large sample of POP patients to determine more representative results. Second, there was a lack of estrogen supplementation in this experiment, and there was a lack of strong evidence to determine whether the screened DEGs and DEMs could be used as therapeutic targets. Therefore, studies need to further investigate the DEGs and DEMs to clarify their potential as therapeutic targets for POP. In addition, this research demonstrated the enrichment of molecular pathways of the DEGs and DEMs, such as the Wnt signaling pathway. However, more cell experiments are needed to confirm whether these pathways play a crucial role in the occurrence of POP caused by long-term menopause.

In summary, we used transcriptome and untargeted metabolome analyses to comprehensively analyze the gene expression profiles and metabolic profiles involved in the development of POP due to prolonged menopause, which leads to decreased vaginal wall support. According to our findings, long-term menopause alters the expression of genes associated with mechanical properties in the vaginal wall and the levels of multiple metabolites involved in various metabolic pathways. In addition, correlation analysis showed that multiple DEGs were substantially associated with DEMs. Together, these studies may provide a new understanding of the underlying mechanisms driving menopausal-induced POP, particularly in exacerbating vaginal wall support injury, and the findings can also be used to target molecular interventions for POP using drug-available gene databases. More research is needed to clarify the mechanism of interaction between differential gene expression in vaginal walls and its associated key metabolites, as well as to evaluate its potential as a therapeutic target for POP.

## Data availability statement

The original contributions presented in the study are publicly available. This data can be found here: https://www.ncbi.nlm.nih.gov/geo/query/acc.cgi?acc=GSE220515.

## Ethics statement

The animal study was reviewed and approved by the ethics committee of the Chengdu Women’s and Children’s Central Hospital.

## Author contributions

ZY and YL: conceptualization, supervision. XY: methodology, writing-original draft preparation, data curation, visualization. LH and WL: methodology, data curation, writing - reviewing and editing. LZ: formal analysis. XZ, BY and YW: methodology, data curation. All authors have read and approved the content of the manuscript.
